# Tri‐modal liquid biopsy: Combinational analysis of circulating tumor cells, exosomes, and cell‐free DNA using machine learning algorithm

**DOI:** 10.1002/ctm2.499

**Published:** 2021-08-04

**Authors:** Jiyoon Bu, Tae Hee Lee, Michael J. Poellmann, Piper A. Rawding, Woo‐Jin Jeong, Rachel S. Hong, Sung Hee Hyun, Hyuk Soo Eun, Seungpyo Hong

**Affiliations:** ^1^ Pharmaceutical Sciences Division School of Pharmacy University of Wisconsin‐Madison Madison Wisconsin USA; ^2^ Wisconsin Center for NanoBioSystems University of Wisconsin‐Madison Madison Wisconsin USA; ^3^ Research Institute for Future Medical Science Chungnam National University Sejong Hospital (CNUSH) Sejong Republic of Korea; ^4^ Department of Senior Healthcare BK21 Plus Program Graduated School Eulji University Daejeon Republic of Korea; ^5^ Department of Biological Sciences and Bioengineering Inha University Incheon Republic of Korea; ^6^ Department of Internal Medicine Chungnam National University School of Medicine Daejeon Republic of Korea; ^7^ Yonsei Frontier Lab and Department of Pharmacy Yonsei University Seoul Republic of Korea

Dear Editor,

Analysis of tumor biomarkers in circulation, commonly known as liquid biopsy, has been highlighted as an effective real‐time monitoring technique for the surveillance of therapeutic responses and tumor progression.^1^, ^2^, ^3^ However, existing liquid biopsy assays that utilize a single tumor biomarker lack the sensitivity and specificity required to obtain clinically reliable information.^4^ In this study, we established a multimodal liquid biopsy (MMLB) system that integrates the expression profiles of the three different tumor biomarkers, circulating tumor cells (CTCs), exosomes, and cell‐free DNA (cfDNA), using a machine learning algorithm (Figure 1).

**FIGURE 1:**
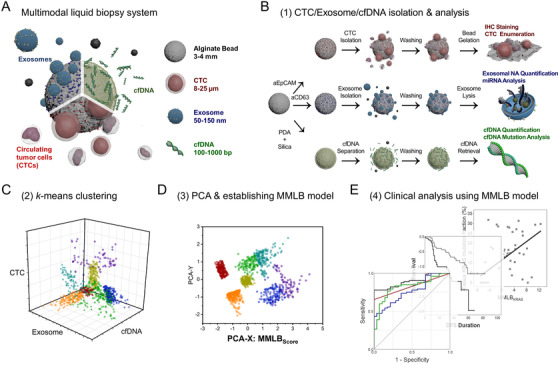
Schematic illustration of the machine learning‐based multimodal analysis of the triple tumor biomarkers – CTCs, exosomes, and cfDNA: (A) A graphical abstract of the multimodal liquid biopsy analysis. (B) Schematic illustration depicting the experimental and analytical procedures for the isolation of CTCs, exosomes, and cfDNA using functionalized alginate beads. (C) Clustering of the CTC counts, exosome NA amounts, and plasma cfDNA concentrations based on the machine learning algorithm; (D) Establishment of MMLB_Score_ for each cohort based on PCA. (E) Validation of the clinical utility of the MMLB analysis

CTCs, exosomes, and cfDNA were isolated using alginate beads functionalized with anti‐epithelial cell adhesion molecule antibodies (aEpCAM), anti‐CD63 antibodies (aCD63), and polydopamine‐silica (PDA‐SiO_2_), respectively. Prior to clinical application, the capture capability of the bead‐based systems was validated using *in vitro* samples (Figure S1). The aEpCAM‐functionalized beads achieved ∼73.1% capture efficiency of EpCAM^+^ SW480 cells, with ∼99.4% leukocyte removal, ∼93.4% cell retrieval, and ∼87.0% cell viability (Figure 2A). Meanwhile, aCD63‐functionalized beads achieved slightly lower recovery of exosome nucleic acids (∼38% less) and PDA‐SiO_2_ beads achieved 1.27‐fold more capture of cfDNA compared with commonly‐used commercial kits (Figures 2B and 2C). Furthermore, all three assays demonstrated high selectivity toward their target biomarkers (Figures 2D–2F).

**FIGURE 2:**
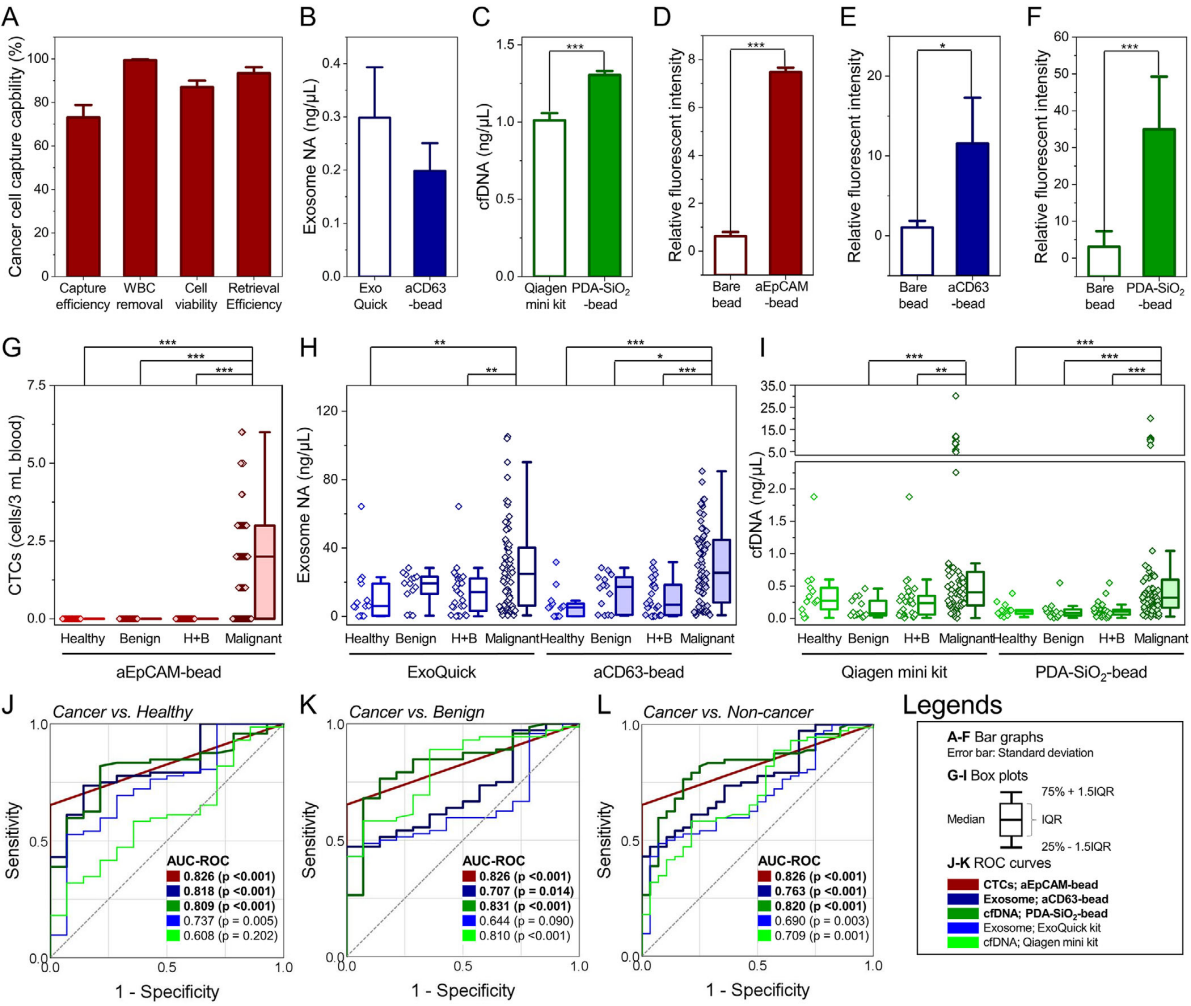
The diagnostic capability of the new bead‐based system for (A) CTCs, (B) exosomes, and (C) cfDNA tested using the human colorectal cancer cell line, SW480 cells. (D‐F) Target specificity of the bead‐based system, validated by comparing CTCs, exosomes, and cfDNA captured on each type of functionalized beads to those captured on bare alginate beads. Note that the capture of all three biomarkers was not prominent on the bare alginate beads, implying that the alginate itself does not contribute to the capture of tumor biomarkers. (G) Number of CTCs, (H) amount of exosome NA, and (I) concentration of plasma cfDNA quantified from a cohort consisting of 72 patients with malignant tumors, 14 patients with benign tumors, and 14 healthy individuals. For exosomes and cfDNA, the bead‐based system was compared with commercially available kits, ExoQuick kit, and Qiagen mini kit, respectively. (J‐L) ROC curves demonstrating the diagnostic capability of the new bead‐based system for distinguishing the patients with malignant tumors from healthy individuals, patients with benign tumors, and overall non‐cancer cohorts (healthy individuals + patients with benign tumors)

The clinical applicability of the beads was investigated using samples obtained from 72 colorectal cancer patients, 14 patients with benign colorectal tumors, and 14 healthy individuals (Table S1). All three systems were capable of differentiating cancer patients from non‐cancer controls, with an AUC‐ROC of 0.826 (CTCs; *p* < 0.001), 0.763 (exosomes; *p* < 0.001), and 0.820 (cDNA; *p* < 0.001), which was greater than commercially available assays (Figures 2G–2L and S2–S5). However, the diagnostic capabilities of the bead‐based systems were still insufficient to be applied in clinical practice due to the inherent variability of each measurement. CTCs were only found in 65.3% of cancer patients, exosomes were elevated in both benign and malignant tumor patients, and cfDNA levels of early‐stage patients were similar to those of the non‐cancer cohorts.

To improve the diagnostic accuracy, *k*‐means clustering was utilized to deduce patterns among the three assays (Figures 3A and 3B). The optimal number of clusters (*k*) was determined to be five based on the elbow method (denoted as A1–A5; Figure S6). Between the clusters, there was a significant difference in the expression profiles of the three biomarkers (Figures 3C–3E). Interestingly, 92.9% (13/14) of healthy individuals and 85.7% (12/14) of patients with benign tumors belonged to cluster A1, which exhibited the lowest expression of all three biomarkers (Table S2). Principle component analysis (PCA) was then used to simplify CTC‐exosome‐cfDNA expressions into a 2D plot (Figure 3F). The x‐axis of the plot (PCA‐X) demonstrated moderate‐to‐strong correlations with all three biomarkers. The PCA‐X was thus defined as MMLB_Score_ which was equivalent to the linear combination of CTCs, exosomes, and cfDNA, with standardized coefficients of 0.600, 0.592, and 0.184, respectively (Figure S7). The MMLB_Score_ was significantly higher among cancer patients (0.40 ± 1.11) compared to non‐cancer cohorts (−1.03 ± 0.33; *p* < 0.001), exhibiting a greater AUC‐ROC (0.894; *p* < 0.001) than any of the single biomarkers (Figures 3G–3K).

**FIGURE 3:**
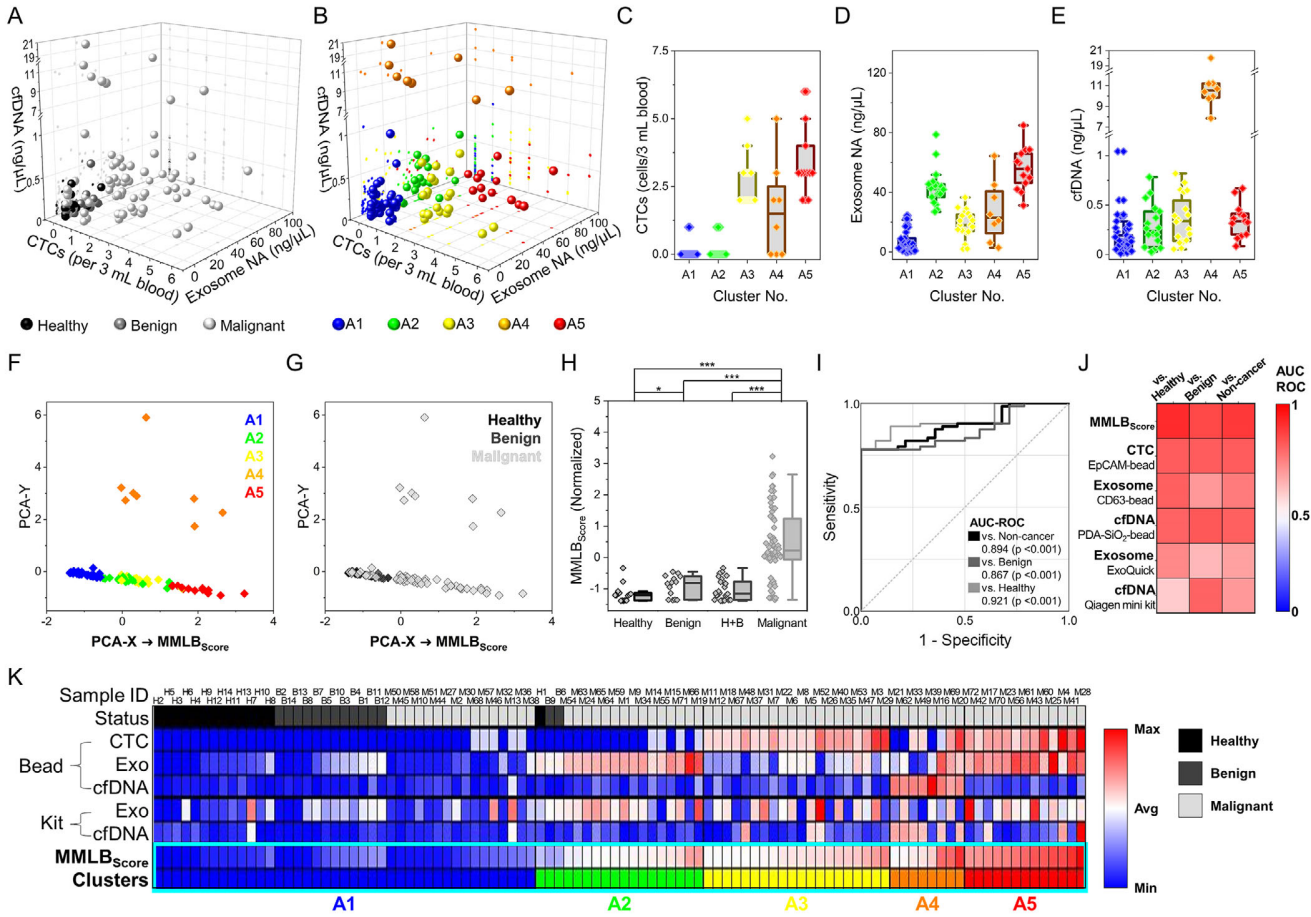
Machine learning‐based clustering of the three tumor biomarkers (CTCs, exosomes, and cfDNA) to establish MMLB_Score_ and validate its diagnostic potential: (A) A 3D scatter plot demonstrating CTC count, exosome NA amount, and plasma cfDNA level of each patient depending on the status (malignant, benign, and healthy) of the cohorts. (B) A *k*‐means clustering of the cohorts based on the expression levels of the three tumor biomarkers. A total of 41, 18, 20, 8, and 13 samples were designated to each cluster, denoted as A1, A2, A3, A4, and A5, respectively. (C‐E) CTC count, exosome NA amount, and plasma cfDNA level for each cluster. (F) PCA applied to reduce the complexity of the 3D plot (CTCs, exosomes, and cfDNA) into the arbitrary 2D plot, with the x‐ and y‐axes consisted of two best linear approximations for stratifying the clusters. The x‐axis in the 2D scatterplot was determined as MMLB_Score_, which minimized the mean‐squared reconstruction error of the clusters and demonstrated a strong correlation with the status of the cohorts. (G and H) MMLB_Score_ depending on the status of the cohorts. (I and J) The ROC curve and a heatmap of AUC‐ROC values demonstrating the enhanced diagnostic capability of MMLB_Score_ compared to any of the single tumor biomarkers used in this study. (K) A heatmap showing expression levels of the three biomarkers compared to MMLB_Score_ for each cohort

We repeated the clustering and PCA on samples from patients with malignant tumors (MMLB_Cancer_) to determine if the score was predictive of pathological status and survival outcomes (Table S3). A strong correlation was found between MMLB_Cancer_ and the T‐stage of a tumor (Figures 4A‐4E and S8), with an AUC‐ROC of 0.761 (*p* < 0.001) for identifying patients with T‐stage ≥ 3. MMLB_Cancer_ outperformed all three biomarkers individually, as well as serum antigens that are routinely tested in clinical practice (Figures 4F and S9). MMLB_Cancer_ also exhibited a moderate correlation with patients’ LVI status, presence of nodal metastasis, and prevalence of distant metastasis (Figures S10 and S11). Furthermore, a Kaplan–Meier survival analysis demonstrated that patients with low MMLB_Cancer_ (≤median) exhibited a 3.36‐fold (*p* = 0.009) longer mean disease‐free survival (DFS) than those with high MMLB_Cancer_ (Table S4), whereas no significant differences were found from the single tumor biomarkers (Figures 4G–4J). Likewise, MMLB_Cancer_ outperformed all three biomarkers for predicting the overall survival (OS) of patients (Figures 4K–4N). As a result, the hazard ratio of MMLB_Cancer_ was 1.370 (*p* = 0.006) and 1.623 (*p* = 0.015) for predicting DFS and OS, respectively, demonstrating superior prediction capabilities in comparison to any of the single biomarkers (Figure 4O).

**FIGURE 4:**
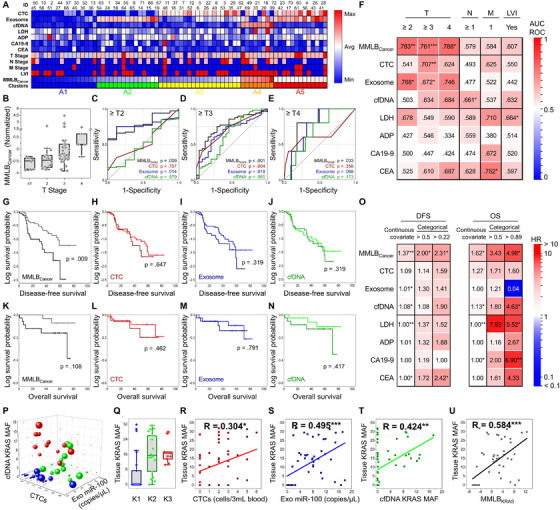
MMLB analysis for determining the pathological features of a tumor, estimating the survival outcomes, and detecting *KRAS* mutation: (A) A heatmap representing MMLB_Cancer_ for each patient, along with their TNM stage, tumor biomarker expressions, and serum antigen expressions. (B) MMLB_Cancer_ for each patient depending on their T stage. (C‐E) ROC curves demonstrating the diagnostic capability of MMLB_Cancer_ (black), CTCs (red), exosomes (blue), and cfDNA (green) for differentiating advanced T stage patients. (F) A heatmap of AUC‐ROC values demonstrating the capability of MMLB_Cancer_ for differentiating patients depending on the pathological features of the tumor, including its size (T stage), the existence of nodal metastasis (N stage), the prevalence of distant metastasis (M stage), and LVI status. (G‐N) Kaplan–Meier survival analysis for (G‐J) DFS and (K‐N) OS between the patients with high (dark) and low (bright) MMLB_Cancer_, CTC counts, exosome NA level, and cfDNA expressions. The median value for each biomarker (or score) was determined as a threshold for dividing the high versus low groups. (O) A heatmap of HR values demonstrating the prognostic capability of MMLB_Cancer_ was compared with the individual tumor biomarkers and serum antigens. (P‐U) The clinical capability of MMLB analysis to determine the tissue *KRAS* mutation. (P) A *k*‐means clustering of patients based on CTC counts, miR‐100 expression in exosomes, and the *KRAS* MAF in cfDNA. The size of the sphere is proportional to the fraction of *KRAS* mutant allele found in tissue. (Q) The *KRAS* MAF in tissue for each cluster. (R‐U) 2D scatterplots representing a correlation with the tissue *KRAS* MAF for MMLB_KRAS_ and the single tumor biomarkers

The MMLB analysis was further applied to detect *KRAS* mutations (MMLB_KRAS_) by combining CTC counts, miR‐100 expression in exosomes, and the fraction of cfDNA KRAS mutant alleles, which have all been reported to be overexpressed in patients with *KRAS* mutation.^5^, ^6^ Clustering analysis revealed that the group which had the lowest expression for all three biomarkers (K1) showed significantly lower tissue *KRAS* mutant allele fraction (∼5.5%) than the other two groups (∼16.9%) (Figures 4P, 4Q, and S12–S15). Furthermore, PCA demonstrated that MMLB_KRAS_ exhibited a strong correlation with tissue *KRAS* mutation status and detected the mutation with high accuracy (Figures 4R–4U and S16).

Our MMLB analysis demonstrated that the utilization of machine learning algorithms has great advantages, as our system was shown to have greater diagnostic/prognostic values than simply adding or multiplying individual tumor biomarkers (Table S5). It should also be noted that the MMLB analysis is not only limited to our bead‐based systems but is also applicable to other liquid biopsy assays. In future studies, improvements will be validated in a larger patient cohort with various tumor types using highly sensitive liquid biopsy assays that our group has developed previously.^7^, ^8^, ^9^, ^10^

In conclusion, we have demonstrated that a machine learning‐based approach that integrates CTC, exosome, and cfDNA liquid biopsy measurements into a single‐score MMLB system was more predictive than each marker alone. This approach may overcome the limitations of existing liquid biopsies with limited sensitivity and specificity. MMLB analysis demonstrated significantly improved accuracy in diagnosing malignancies, determining the pathological status of patients, predicting survival outcomes, and detecting gene mutations. These findings suggest that our approach markedly enhances liquid biopsy assays to ultimately achieve personalized medicine and improve patient outcomes.

## AUTHOR CONTRIBUTIONS

Seungpyo Hong, Jiyoon Bu, and Tae Hee Lee designed the concept. Tae Hee Lee and Sung Hee Hyun conducted experiments. Jiyoon Bu and Tae Hee Lee performed the statistical analysis. Piper A. Rawding, Michael J. Poellmann, Rachel S. Hong, and Woo‐Jin Jeong reviewed the analysis. Jiyoon Bu, Seungpyo Hong, Piper A. Rawding, and Rachel S. Hong wrote the manuscript. Tae Hee Lee, Piper A. Rawding, Woo‐Jin Jeong, Michael J. Poellmann, and Sung Hee Hyun revised the manuscript.

## AVAILABILITY OF DATA AND MATERIALS

All data generated from this study are included in this published article and supporting information. Raw data are available from the corresponding author on reasonable request.

## ETHICS APPROVAL AND CONSENT TO PARTICIPATE

Informed consent was obtained in accordance with the Declaration of Helsinki. This study was approved by the Ethics Committee of Eulji University (study numbers: EU 2017–44 and EU2018‐68)

## Supporting information

Supporting Information
