# Supplementary material for: Tri‐modal liquid biopsy: Combinational analysis of circulating tumor cells, exosomes, and cell‐free DNA using machine learning algorithm
Source: Clin Transl Med. 2021 Aug 4;11(8):e499. doi: 10.1002/ctm2.499 (PMC8335965; doi:10.1002/ctm2.499)
Supplement: Supplementary file 1 — Supporting Information [file CTM2-11-e499-s001.docx]

*Supplementary Materials*

**Tri-modal Liquid Biopsy: Combinational analysis of circulating tumor cells, exosomes, and cell-free DNA using machine learning algorithm**

*Jiyoon Bu^1,2†^, Tae Hee Lee^3,4†^, Michael J. Poellmann,^1,2^ Piper A. Rawding^1,2^, Woo-jin Jeong^1,5^, Rachel S. Hong^1^, Sung Hee Hyun^4^, Hyuk Soo Eun^6^, and Seungpyo Hong^1,2,7*^*

^1^ Pharmaceutical Sciences Division, School of Pharmacy, University of Wisconsin-Madison, Madison, WI 53705, USA
E-mail: seungpyo.hong@wisc.edu

^2^ Wisconsin Center for NanoBioSystems, University of Wisconsin-Madison, Madison, WI 53705, USA

^3^ Research Institute for Future Medical Science, Chungnam National University Sejong Hospital (CNUSH), Sejong 30099, Republic of Korea

^4^ Department of Senior Healthcare, BK21 plus program, Graduated School, Eulji University, Daejeon 34824, Republic of Korea

^5^ Department of Biological Sciences and Bioengineering, Inha University, 100 Inha-ro, Michuhol-gu, Incheon 22212, Republic of Korea

^6^ Department of Internal Medicine, Chungnam National University School of Medicine, Daejeon 35015, Republic of Korea

^7^ Yonsei Frontier Lab and Department of Pharmacy, Yonsei University, Seoul 03722,

Republic of Korea

^†^ These authors contributed equally to this work

*Address all correspondence to:

Prof. Seungpyo Hong

Pharmaceutical Sciences Division, School of Pharmacy

University of Wisconsin – Madison

7121 Rennebohm Hall

777 Highland Avenue

Madison, WI 53705, USA

email: seungpyo.hong@wisc.edu

phone: (608) 890-0699

**Methods**

**Materials**

Sodium alginate, calcium chloride (CaCl_2_), 1-(3-Dimethylaminopropyl)-3-EthylcarbodiimideHydrochloride (EDC), N-Hydroxy-Succinimide (NHS), silica solution (LUDOX® AM colloidal silica, 30 wt. % in H_2_O), and 2-(3,4-Dihydroxyphenyl) ethylamine hydrochloride (dopamine hydrochloride), were obtained from Sigma–Aldrich (St. Louis, MO, USA). Ethylenediaminetetraacetic acid (EDTA) and Trizol® were purchased from Invitrogen (Carlsbad, CA, USA). Tris (hydroxymethyl) aminomethane hydrochloride (Tris-HCl) was obtained from Millipore (Billerica, MA). CD326 (EpCAM) Monoclonal Antibody (aEpCAM) was purchased from eBioscience™ (San Diego, CA, USA) and anti-CD63 antibody (aCD63) was purchased from Abcam (Cambridge, UK). ExoQuick was purchased from System Biosciences (Palo Alto, CA, USA). Cell culture media RPMI-1640 medium, fetal bovine serum (FBS), and penicillin-streptomycin (P/S)were all purchased from Invitrogen Corporation (Carlsbad, CA). The QIAmp DNA Mini Kit was purchased from Qiagen Inc (Qiagen, Hilden, Germany).

**Cell Culture**

A human colorectal adenocarcinoma cancer cell line, SW480, was purchased from the Korean Cell Line Bank (Seoul, Republic of Korea) on Mar. 06, 2020. Cells were frozen in aliquots at passage three and stored in liquid nitrogen. All *in vitro* experiments were conducted after three passages post-thaw, up to 6 months from the purchase. Cells were grown up to 80% confluency under a humidified condition at 37°C, 5% CO_2_ in RPMI-1640 medium supplemented with 10% (v/v) FBS and 1% (v/v) P/S. The *in vitro* efficiencies were obtained from at least three times of trials.

**Clinical Samples**

This study was approved by an institutional review board (IRB) of Eulji University, Daejeon, Korea (EU17-44 and 18-68). The blood samples were collected from the National University Hospital Biobank of Chungbuk. The samples were prepared in BC Vacutainer tubes containing EDTA to prevent the coagulation. The cohorts consist of 72 colorectal cancer patients of any age, identified at the National University Hospital Biobank of Chungbuk, 14 patients with benign colorectal tumors, and 14 healthy individuals. The median age of each group was 67 (interquartile range, 58–71) years, 56 (38–63) years, and 55 (52–61) years old for patients with colorectal cancer, patients with benign tumor, and healthy individuals, respectively. The median weight of each group was 58.5 (55.75 - 61.25) kg, 66.4 (59.25 - 71.75) kg, and 64 (57 - 66) kg. The clinical information of the patients, including age, gender, TNM stage, LVI status, survival outcomes, and serum antigen levels, were also given from the National University Hospital Biobank of Chungbuk, which was determined by the physical exam, computed tomography, biopsy, and/or serum antigen tests. Patients’ information was blinded until expression profiles of CTCs, exosomes, and cfDNA was quantified. Of the 72 patients, 17 were excluded for the *KRAS* mutation analysis whose sum of *KRAS* mutant and wild type was less than 30 copies/µL from the biopsy specimen. Buffy coats and plasma were separated from 3 mL blood as described previously.^26^

**Statistics**

The differences in the amount of each tumor biomarker expression (or MMLB_Score_) between the subgroups were examined using Student’s t-test or Mann-Whitney U test, depending on the normality of the data. ROC analysis was conducted to compare the diagnostic/prognostic capabilities of these biomarkers for determining the patients with the malignant tumor or advanced TNM stages. The survival analysis was conducted using the Kaplan-Meier plots and the Cox Regression model for both overall survival (OS) and disease-free survival (DFS). All P values less than 0.05, 0.01, and 0.001 were denoted with one, two, and three asterisks, respectively.

**Bead preparation**

*Alginate bead synthesis*

Alginate beads were prepared through ionic crosslinking of the hydrogel. Briefly, alginate in solution (5% in w/v) was added dropwise into deionized water (DW) containing calcium chloride (100 mM) using 200 µL pipette and stirred gently at room temperature for 1 h. Alginate was cross-linked to beads, taken out from the solution, washed with DW, and stored in DW at 4˚C until further use.

*EDC/NHS reaction on alginate beads*

The carboxylic groups on the alginate beads were activated using EDC/NHS chemistry. The beads were treated with 200 mM EDC and 200 nM NHS in ddH_2_O at room temperature for 1 h. The beads were immobilized with either aEpCAM, aCD63, or PDA-SiO_2_ for the capture of CTCs, exosomes, and cfDNA, respectively.

*Immobilization of aEpCAM, aCD63, or PDA-SiO_2_*

For preparing aEpCAM- or aCD63-functionalized beads, NHS-activated beads were incubated in PBS solution containing either aEpCAM (1% v/v) or aCD63 (1% v/v) for 10 min at room temperature and an additional 1 h at 4˚C. For the PDA-SiO_2_-immobilized beads, alginate beads were substantially reacted with dopamine hydrochloride and silica using EDC/NHS chemistry, as demonstrated previously.^26^

***In Vitro* Sample Preparation**

*Cell preparation*

Cells were washed with PBS three times and detached from the T flask using trypsin-EDTA. The surface-detached cells were collected using centrifugation at 200 g for 3 min and resuspended in 1 mL of complete media.

*Cell supernatant preparation*

After cells were grown up to 80% confluency, cell culture media were replaced with the free RPMI-1640 media. Cells were culture for 24 h and the media was collected from the T flask. The supernatant was collected from the media using centrifugation at 200 g for 3 min, followed by the removal of the cell pellets. The centrifugation was repeated three times to get rid of the cells and large cellular debris.

**Isolation of CTCs (or cancer cells)**

*Capture and release of CTCs (or cancer cells) using aEpCAM-functionalized beads*

aEpCAM-functionalized beads were incubated for an hour with a gentle stirring in 2 mL of diluted buffy coat layer which consisted of 1 mL PBS mixed with 1 mL buffy coat layer obtained from 3 mL human blood (or 1 mL of cell media containing ~3 × 10^4^ cells for *in vitro* samples). After the cell capture, the beads were taken out from the buffy coats, followed by three times wash with PBS. The cells captured on the beads were released by incubating the beads with PBS-EDTA (0.5 M, pH 8.0) for 10 min.

*Determination of the capture efficiency, retrieval efficiency, WBC removal, and viability of the capture cancer cells*

For *in vitro* samples, capture efficiency, retrieval efficiency, leukocyte removal, and viability were determined as follows: The capture efficiency was defined as the ratio of the number of cancer cells captured on aEpCMA-functionalized beads compared to the initial number of cancer cells (~3 × 10^4^ cells) incubated with the beads. The number of cancer cells captured on the beads was determined by subtracting the number of cells that remained in the solution after an hour of incubation to the initial number of cells. The retrieval efficiency was defined as the ratio of the number of cancer cells released from the EpCAM-functionalized beads upon incubation in EDTA-PBS, compared to the number of cells that have been captured on the bead surface. Leukocyte removal was calculated in a similar way that we measured the cancer cell capture efficiency. The ratio of leukocytes that were unbound to the beads was determined as a leukocyte removal rate. Note that the initial number of leukocytes was ~1.5 × 10^6^ cells, which correspond to the number of leukocytes obtained from 3 mL of whole blood. The viability of the cancer cells after their capture and release from the beads was determined using toluidine blue assay.

*Immunohistochemistry*

For clinical samples, the cells released from the beads were attached to the glass slide upon cytospin at 1,500 rpm, for 3 min. Cells were immediately fixed with 4% paraformaldehyde for 15 min and subsequently incubated in an antigen retrieval buffer solution (10mM Sodium Citrate, 0.05% Tween 20, pH 6.0) for 60 min, which was pre-heated to 90°C. The slides were then cooled for 10 min at room temperature. After the inactivation of endogenous peroxidase, the slides were incubated in 1% BSA to prevent the non-specific bindings of the antibodies. Cells were then treated with aEpCAM (TS63, Abcam; 1:200 in PBS) for an hour, washed with wash buffer (DAKO) three times, and reacted with DakoREAL EnVision detection reagent (Dako) for 40 min. The cells were again washed with the wash buffer (DAKO) three times and incubated with DAB reagent (3,3-diaminobenzidine tetrahydrochloride) (DAKO) for 10 min, followed by another three-time wash with DW and counterstaining with Meyer’s hematoxylin. EpCAM-positive CTCs were determined as follows: 1) The cells having unequivocal brownish staining (aEpCAM) in the cell membrane, 2) having a diameter larger than 12 µm, and 3) having a higher N/C ratio compared to other background leukocytes.

**Isolation of exosomes**

*The capture of exosomes using aCD63-functionalized beads*

aCD63-functionalized beads were incubated in either 250 µL cell-free plasma or cell-removed supernatant at room temperature for an hour with gentle stirring. Beads were taken out from the plasma, followed by three times wash with PBS. The exosomes captured on the beads were released by incubating the beads with PBS-EDTA (0.5 M, pH 8.0) for 10 min.

*The capture of exosomes using ExoQuick*

The same amount of cell-free plasma or cell-removed supernatant samples (250 µL) was mixed with 63 µL of ExoQuick™ solution and incubated for 30 min at 4˚C. The sample-exosome mixture was then centrifuged at 1,500 g for 10 min, followed by the removal of the supernatant. The exosome pellets were re-suspended in PBS.

*Exosome NA extraction and Quantification*

TRIzol reagents (500 µL) were added to the samples to extract RNA from exosomes. Samples were mixed by pipetting for 3 min and incubated for an additional 5 min at room temperature. After the incubation, 100 µL chloroform was added and mixed thoroughly for 15 s using the shaker, followed by 10 min incubation at room temperature. Phase separation was then performed upon centrifugation (13,000 rpm at 4 ˚C for 10 min). The upper aqueous phase was collected and mixed with 500 µL of isopropanol. The samples were vortexed and incubated at room temperature for 10 minutes. The samples were centrifuged once more at 13,000 rpm at 4 ˚C for 10 min. The supernatants were aspirated and 600 µL of 70 % ethanol was added to remove phenol. Ethanol was then removed by centrifugation at 13,000 rpm at 4 ˚C for 5 min, followed by air-drying the exosome NA pellet for 5 min. The pellets were re-suspended to 50 µL of DW. The concentration and integrity of exosome NA was determined using Experion™ automated electrophoresis system (Bio-Rad Laboratories, Munich, Germany) with RNA HighSens Chips.

**Isolation of cfDNA**

*The capture of cfDNA using PDA-SiO_2_-functionalized beads*

PDA-SiO_2_-functionalized beads were incubated in either 200 µL cell-free plasma or cell-removed supernatant and treated with proteinase K at a 10:1 (v/v) ratio. The samples were further mixed with 200 lysis buffer and incubated at 37 ˚C for 10 min, followed by the reaction with 200 μL of 95% ethanol. A PDA-SiO_2_-functionalized bead was then added into the sample with 5 μL calcium chloride solution, followed by incubation under gentle agitation for 10 min, allowing the adsorption of cfDNA onto the bead surface. The beads were washed with AW1 wash buffer and stored in RNase/DNase free water.

*The capture of cfDNA using QIAmp DNA mini kit*

QIAamp DNA mini kit was also employed for the DNA extraction and compared with our system, following the manufacturers’ instructions. The details can be found in our earlier publication.^1^

*cfDNA quantification*

The concentration of plasma cfDNA was quantified using Agilent Bioanalyzer 2100 instrument and the High Sensitivity DNA Kit (Agilent Technologies, CA, USA), following the manufacturer’s recommended protocol. The amount of DNA between 100 – 1000 bp was quantitatively assessed. The details can also be found in our earlier publication.^1^

**Droplet digital polymerase chain reaction (ddPCR)**

*DNA extraction from tissue*

For extraction of genomic DNA from the tissue, 200 μL lysis buffer was added to the frozen tissue fragment. The tissue sample was placed on a pestle which was kept on ice and pulverized using mortar. Samples were then centrifuged at 3,000 rpm for 10 min and the supernatants were extracted using PDA-SiO_2_-functionalized beads, as aforementioned. Samples were stored at -70˚C until processed.

*ddPCR for KRAS^G12/G13^*

ddPCR was conducted using QX200 Droplet Digital PCR System (Bio-Rad, Hercules, CA) and the *KRAS*^G12/G13^ screening multiplex kit (Bio-rad) was utilized to quantify the *KRAS* mutation from tissue-derived DNA and cfDNA. Samples (1 µL; 5× concentrated from serum) were reacted with 10 μL of 2× ddPCR Supermix for probes (Bio-Rad), 1 μL of 20× Multiplex Screening Probes (Bio-Rad), and 8 μL of RNase/DNase free water and loaded into a disposable droplet generator cartridge. Droplet generator oil for probes (70 μL; Bio-Rad) was added to each well and the cartridge was placed inside the QX200 droplet generator (Bio-Rad). The droplets were generated and transferred to a 96-well PCR plate. The plate was subsequently heat-sealed with foil and PCR was performed on a thermal cycler as follows: 95 ℃ for 10 min (1 cycle); 94 ℃ for 30 s and 55 ℃ for 1 min (40 cycles); 98 ℃ for 10 min (1 cycle); 4 ℃ holds. The plate was then transferred to QX200 droplet reader (Bio-Rad) for fluorescence measurement of mutant probe labeled with 6‐fluorescein amidite (FAM) and wild type probe labeled with hexachloro‐fluorescein (HEX) channels. The QuantaSoft^TM^ Analysis Pro software was employed to determine the amount of the target DNA in copies/µL. The mutant allele frequency (MAF) was calculated by dividing mutant type droplets by the total (mutant + wild type) droplets.

*ddPCR for miR-100*

Complementary DNA (cDNA) for the detection of miR-100 was synthesized using ReverTra Ace® qPCR RT Master Mix with gDNA Remover (Toyobo, Osaka, Japan), according to the manufacturer’s instructions. Briefly, exosome-extracted NA (1 μL) was incubated at 65 ℃ for 5 min, followed by adding 2 μL of 4× DN master mix and 5 μL of Nuclease-free water. The reagent was incubated at 37℃ for 5 min. 5× RT Master mix Ⅱ (2 µL) was added and incubated at 37℃ for 5 min (1 cycle); at 50℃ for 5 min (1 cycle), then, reacted at 90℃ for 5 min. The cDNA (1 μL) was then mixed with 2× ddPCR Supermix for probes (10 μL), hsa-miR-100-5p (assay ID, 478224, 2 μL), and DW (7 μL). ddPCR was conducted following the same procedures that we have done for *KRAS* mutation analysis, while there was slight difference in the thermal cycle: 95 ℃ for 10 min (1 cycle); 95 ℃ for 30 s and 57 ℃ for 1 min (40 cycles); 4 ℃ for 5 min (1 cycle); 98 ℃ for 10 min (1 cycle); 4 ℃ holds.

**Machine learning-based analysis**

*k-means clustering*

A *k*-means clustering algorithm was utilized to obtain the clusters from a data set obtained from each of tumor biomarker analyses. The number of clusters for each analysis was determined based on the elbow method. The range of clusters was between 2 and 14.

**Tables**

**Table S1**. The number of CTCs, amount of exosome NA, and concentration of plasma cfDNA obtained from healthy individuals, patients with benign tumors, and patients with malignant tumors. MMLB_Score_ was acquired by integrating the amount of the three biomarkers obtained from our bead-based assay, based on the machine learning algorithm. The numbers in each cell denote median (IQR) unless otherwise stated.

|  | Total n=100 | Healthy n = 14 | Benign n = 14 | Malignant n = 72 |
| --- | --- | --- | --- | --- |
| *Age* | 63 (55 - 70) | 55 (52.25 - 60.5) | 55.5 (38 - 63) | 67 (58 - 71) |
| *Gender (F/M)* | 40/60 | 13/1 | 3/11 | 24/48 |
| *CTC* |  |  |  |  |
| counts | 0 (0 - 2) | 0 (0 - 0) | 0 (0 - 0) | 2 (0 - 3) |
| ≥1 | 47 (47%) | 0 (0%) | 0 (0%) | 47 (65.28%) |
| *Exosome* |  |  |  |  |
| bead | 18.84 (5.04 - 39.35) | 5.24 (0.15 - 7.12) | 17.28 (2.58 - 22.59) | 25.5 (8.53 - 44.6) |
| kit | 20.25 (5.99 - 32.58) | 6.09 (1.63 - 16.75) | 19.31 (13.7 - 23.05) | 24.85 (6.43 - 40.23) |
| *cfDNA* |  |  |  |  |
| bead | 0.21 (0.11 - 0.41) | 0.12 (0.08 - 0.13) | 0.09 (0.05 - 0.13) | 0.32 (0.17 - 0.59) |
| kit | 0.34 (0.14 - 0.59) | 0.27 (0.17 - 0.46) | 0.08 (0.05 - 0.26) | 0.41 (0.2 - 0.72) |
| *MMLB_Score_* | -0.02 (-1.07 - 0.56) | -1.2 (-1.37 - -1.14) | -0.82 (-1.29 - -0.62) | 0.23 (-0.05 - 1.24) |

CTC counts (cells/ 3 mL whole blood); exosomes (ng/µL); cfDNA (ng/µL); MMLB_Score_ (normalized).

**Table S2**. The number of CTCs, amount of exosome NA, and concentration of plasma cfDNA in each cluster.

| All cohorts | A1 n = 41 | A2 n = 18 | A3 n = 20 | A4 n = 8 | A5 n = 13 |
| --- | --- | --- | --- | --- | --- |
| *CTC* |  |  |  |  |  |
| mean ± SD | 0.12 ± 0.33 | 0.22 ± 0.43 | 2.7 ± 0.98 | 1.63 ± 1.77 | 3.46 ± 1.39 |
| median (IQR) | 0 (0–0) | 0 (0–0) | 2 (2–3) | 1.50 (0–2.25) | 3 (3–4) |
| *Exosome* |  |  |  |  |  |
| mean ± SD | 7.19 ± 7.58 | 43.51 ± 12.5 | 18.53 ± 9.1 | 27.51 ± 20.45 | 55.78 ± 14.42 |
| median (IQR) | 4.93 (0.93–9.25) | 42.3 (37.43–45.43) | 18.1 (14.2–24.68) | 23.1 (15.73–38.68) | 55.8 (46.3–66) |
| *cfDNA* |  |  |  |  |  |
| mean ± SD | 0.17 ± 0.18 | 0.28 ± 0.22 | 0.35 ± 0.24 | 11.4 ± 3.65 | 0.35 ± 0.17 |
| median (IQR) | 0.12 (0.07–0.19) | 0.24 (0.09–0.42) | 0.34 (0.14–0.5) | 10.56 (9.92–11.2) | 0.34 (0.20–0.41) |
| *MMLB_Score_* |  |  |  |  |  |
| mean ± SD | -1.09 ± 0.26 | 0.15 ± 0.47 | 0.42 ± 0.45 | 0.98 ± 1.02 | 1.97 ± 0.55 |
| median (IQR) | -1.16 (-1.30–-0.84) | 0.08 (-0.08–0.25) | 0.36 (0.05–0.64) | 0.5 (0.24–1.9) | 1.92 (1.57–2.27) |
| Cancer Patients | A1 n = 16 | A2 n = 15 | A3 n = 20 | A4 n = 8 | A5 n = 13 |
| *CTC* |  |  |  |  |  |
| mean ± SD | 0.31 ± 0.48 | 0.27 ± 0.46 | 2.7 ± 0.98 | 1.63 ± 1.77 | 3.46 ± 1.39 |
| median (IQR) | 0 (0–1) | 0 (0–0.5) | 2 (2–3) | 1.50 (0–2.25) | 3 (3–4) |
| *Exosome* |  |  |  |  |  |
| mean ± SD | 5.38 ± 6.1 | 46.41 ± 11.62 | 18.53 ± 9.1 | 27.51 ± 20.45 | 55.78 ± 14.42 |
| median (IQR) | 2.75 (2.48–5.98) | 44.0 (40.0–46.3) | 18.1 (14.2–24.68) | 23.1 (15.73–38.68) | 55.8 (46.3–66) |
| *cfDNA* |  |  |  |  |  |
| mean ± SD | 0.23 ± 0.24 | 0.32 ± 0.22 | 0.35 ± 0.24 | 11.4 ± 3.65 | 0.35 ± 0.17 |
| median (IQR) | 0.17 (0.08–0.28) | 0.27 (0.17–0.45) | 0.34 (0.14–0.5) | 10.56 (9.92–11.2) | 0.34 (0.20–0.41) |
| *MMLB_Cancer_* |  |  |  |  |  |
| mean ± SD | -0.78 ± 0.1 | -0.26 ± 0.16 | -0.32 ± 0.14 | 2.49 ± 0.86 | 0.22 ± 0.18 |
| median (IQR) | -0.82 (-0.84–-0.72) | -0.3 (-0.36–-0.26) | -0.35 (-0.41–-0.23) | 2.10 (2–2.67) | 0.21 (0.08–0.37) |

CTC counts (cells/ 3 mL whole blood); exosomes (ng/µL); cfDNA (ng/µL); MMLB_Score_ (normalized).

**Tables S3**. Clinico-pathological characteristics of enrolled cancer patients depending on the cluster.

|  | A1 n = 16 | A2 n = 15 | A3 n = 20 | A4 n = 8 | A5 n = 13 |
| --- | --- | --- | --- | --- | --- |
| T Stage |  |  |  |  |  |
| in situ | 0 (0%) | 0 (0%) | 2 (10%) | 0 (0%) | 0 (0%) |
| T1 | 3 (18.8%) | 0 (0%) | 3 (15%) | 0 (0%) | 0 (0%) |
| T2 | 7 (43.8%) | 5 (33.3%) | 3 (15%) | 1 (12.5%) | 0 (0%) |
| T3 | 6 (37.5%) | 9 (60%) | 12 (60%) | 6 (75%) | 10 (76.9%) |
| T4 | 0 (0%) | 1 (6.7%) | 0 (0%) | 1 (12.5%) | 3 (23.1%) |
| N Stage |  |  |  |  |  |
| N0 | 11 (68.8%) | 8 (53.3%) | 11 (55%) | 2 (25%) | 9 (69.2%) |
| N1 | 3 (18.8%) | 5 (33.3%) | 5 (25%) | 3 (37.5%) | 4 (30.8%) |
| N2 | 2 (12.5%) | 2 (13.3%) | 4 (20%) | 3 (37.5%) | 0 (0%) |
| M Stage |  |  |  |  |  |
| M0 | 15 (93.8%) | 13 (86.7%) | 19 (95%) | 7 (87.5%) | 10 (76.9%) |
| M1 | 1 (6.3%) | 2 (13.3%) | 1 (5%) | 1 (12.5%) | 3 (23.1%) |
| LVI |  |  |  |  |  |
| Yes | 9 (56.3%) | 9 (60%) | 11 (55%) | 0 (0%) | 6 (46.2%) |
| No | 7 (43.8%) | 6 (40%) | 9 (45%) | 8 (100%) | 7 (53.8%) |

**Table S4**. Univariate HR for DFS and OS. The numbers in each cell denote HR (95% CI).

| Marker | DFS | | | | OS | | |
| --- | --- | --- | --- | --- | --- | --- | --- |
|  | Continuous covariate | Threshold  > Median | Threshold  > 22nd Per. | Continuous covariate | | Threshold  > Median | Threshold  > 89th Per. |
| MMLB | 1.370  (1.093–1.717)  p = 0.006 | 1.997  (1.165–3.422)  p = 0.012 | 2.313  (1.122–4.768)  p = 0.023 | 1.623  (1.100–2.395)  p = 0.015 | | 3.432  (0.692–17.028)  p = 0.131 | 4.982  (1.182–21.004)  p = 0.029 |
| CTC | 1.089  (0.933–1.272)  p = 0.279 | 1.140  (0.643–2.020)  p = 0.655 | 1.585  (0.892–2.818)  p = 0.116 | 1.269  (0.833–1.932)  p = 0.267 | | 1.705  (0.405–7.184)  p = 0.467 | 1.602  (0.193–13.308)  p = 0.663 |
| Exosome | 1.013  (1–1.026)  p = 0.047 | 1.300  (0.766–2.206)  p = 0.331 | 1.405  (0.725–2.724)  p = 0.314 | 0.992  (0.959–1.026)  p = 0.646 | | 1.209  (0.296–4.936)  p = 0.792 | 0.042  (0–899.881)  p = 0.533 |
| cfDNA | 1.076  (1.008–1.148)  p = 0.028 | 1.076  (0.637–1.819)  p = 0.784 | 1.902  (0.957–3.779)  p = 0.067 | 1.125  (1.022–1.237)  p = 0.016 | | 1.796  (0.428–7.529)  p = 0.423 | 4.627  (1.097–19.509)  p = 0.037 |
| LDH | 1.001  (1–1.002)  p = 0.002 | 1.373  (0.811–2.325)  p = 0.239 | 1.518  (0.784–2.94)  p = 0.216 | 1.001  (1–1.002)  p = 0.007 | | 7.925  (0.967–64.944)  p = 0.054 | 5.518  (1.310–23.247)  p = 0.02 |
| ADP | 1.005  (0.997–1.014)  p = 0.242 | 1.320  (0.781–2.231)  p = 0.3 | 1.877  (0.946–3.724)  p = 0.072 | 0.998  (0.974–1.024)  p = 0.903 | | 1.162  (0.29–4.653)  p = 0.832 | 2.672  (0.534–13.356)  p = 0.231 |
| CA19-9 | 1  (0.999–1.002)  p = 0.881 | 1.194  (0.705–2.021)  p = 0.51 | 0.998  (0.527–1.891)  p = 0.995 | 1.002  (1–1.004)  p = 0.019 | | 2.001  (0.474–8.451)  p = 0.345 | 6.898  (1.619–29.398)  p = 0.009 |
| CEA | 1.002  (1–1.004)  p = 0.044 | 1.729  (1.013–2.951)  p = 0.045 | 2.418  (1.177–4.969)  p = 0.016 | 1.001  (0.996–1.006)  p = 0.637 | | 1.605  (0.383–6.731)  p = 0.518 | 4.329  (0.824–22.758)  p = 0.083 |

**Table S5**. Diagnostic/prognostic capability of machine learning-based MMLB anlaysis compared to simply adding or multiplying the amount of CTCs, exosomes, and cfDNA obtained from the bead assays.

| Marker | AUC-ROC | | | | | | HR | |
| --- | --- | --- | --- | --- | --- | --- | --- | --- |
|  | Malignant  Tumor | Large  A tumor (T4) | Nodal  Meta. | Distant  Meta. | LVI | *KRAS* mutation | DFS | OS |
| MMLB | 0.894  (0.863–0.979) p <0.001 | 0.788  (0.639–0.937)  p = 0.033 | 0.579  (0.445–0.714)  p = 0.253 | 0.584  (0.367–0.801)  p = 0.441 | 0.607  (0.475–0.739)  p = 0.119 | 0.951  (0.864 –1.000)  p <0.001 | 1.370  (1.093–1.717)  p = 0.006 | 1.623  (1.1–2.395)  p = 0.015 |
| Add. | 0.894  (0.861–0.978) p <0.001 | 0.761  (0.558–0.964)  p = 0.053 | 0.558  (0.423–0.693)  p = 0.403 | 0.578  (0.347–0.809)  p = 0.474 | 0.608  (0.475–0.740)  p = 0.116 | 0.906  (0.830–0.981)  p <0.001 | 1.196  (1.046–1.367)  p = 0.009 | 1.425  (0.997–2.036)  p = 0.052 |
| Mult. | 0.893  (0.861 - 0.978) p <0.001 | 0.74  (0.514–0.966)  p = 0.075 | 0.506  (0.370–0.641)  p = 0.932 | 0.516  (0.277–0.755)  p = 0.886 | 0.557  (0.422–0.691)  p = 0.408 | 0.890  (0.806–0.974)  p <0.001 | 1.022  (0.960–1.088)  p = 0.497 | 1.033  (0.860–1.242)  p = 0.727 |
| CTC | 0.826  (0.74–0.913)  p <0.001 | 0.624  (0.287–0.96)  p = 0.358 | 0.493  (0.358–0.629)  p = 0.923 | 0.625  (0.398–0.852)  p = 0.251 | 0.55  (0.416–0.684)  p = 0.464 | 0.622  (0.475–0.769)  p = 0.135 | 1.089  (0.933–1.272)  p = 0.279 | 1.269  (0.833–1.932)  p = 0.267 |
| Exosome | 0.763  (0.714–0.923) p <0.001 | 0.746  (0.597–0.895)  p = 0.068 | 0.477  (0.341–0.613)  p = 0.737 | 0.522  (0.286–0.759)  p = 0.837 | 0.442  (0.307–0.577)  p = 0.398 | 0.949  (0.858–1.000)  p = 0 | 1.013  (1–1.026)  p = 0.047 | 0.992  (0.959–1.026)  p = 0.646 |
| cfDNA | 0.820  (0.703–0.914) p <0.001 | 0.684  (0.497–0.87)  p = 0.173 | 0.661  (0.535–0.787)  p = 0.02 | 0.537  (0.335–0.739)  p = 0.734 | 0.632  (0.503–0.762)  p = 0.053 | 0.749  (0.623–0.874)  p = 0.002 | 1.076  (1.008–1.148)  p = 0.028 | 1.125  (1.022–1.237)  p = 0.016 |

Add. = Z_CTC_ + Z_Exosome_ + Z_cfDNA_

Multi. = (Z_CTC_ + min_(CTC,Exosome,cfDNA)_) × (Z_Exosome_ + min_(CTC,Exosome,cfDNA)_)

× (Z_cfDNA_ + min_(CTC,Exosome,cfDNA)_)

**Figures**


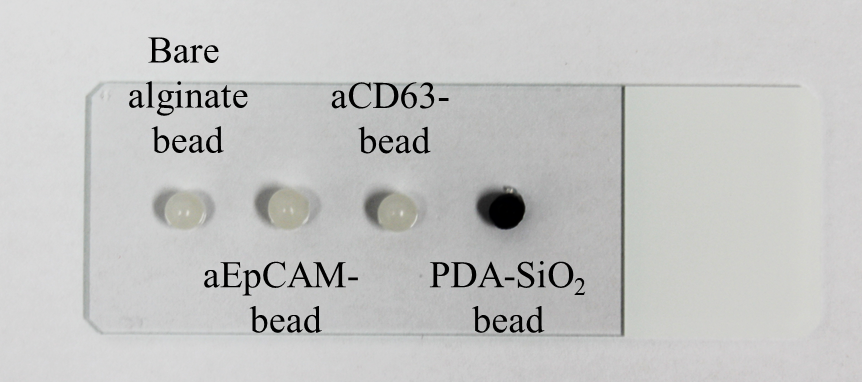


**Figure S1**. Image of the aEpCAM-functionalized, aCD63-functionalized, and PDA-SiO_2_-functionalized beads for isolating CTCs, exosomes, and cfDNA, respectively.


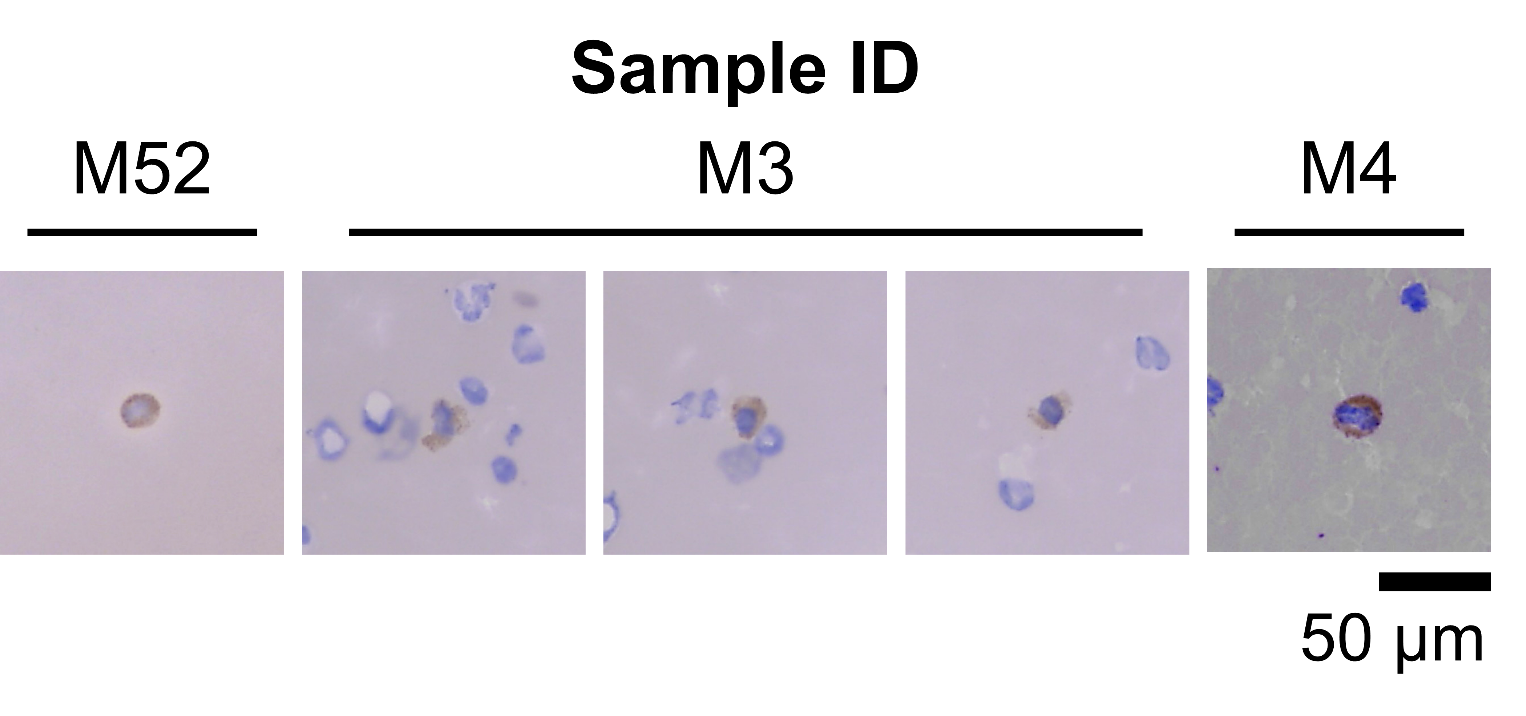


**Figure S2**. Representative IHC image of captured CTCs. CTC-positivity rate of 65.3% (47/72) for cancer patients, with an average of 1.68 ± 1.64 CTCs per 3 mL blood (Figure 2G and S2). Meanwhile, CTCs were not detected from any of the samples obtained from healthy individuals or patients with benign tumors (p <0.001).

**
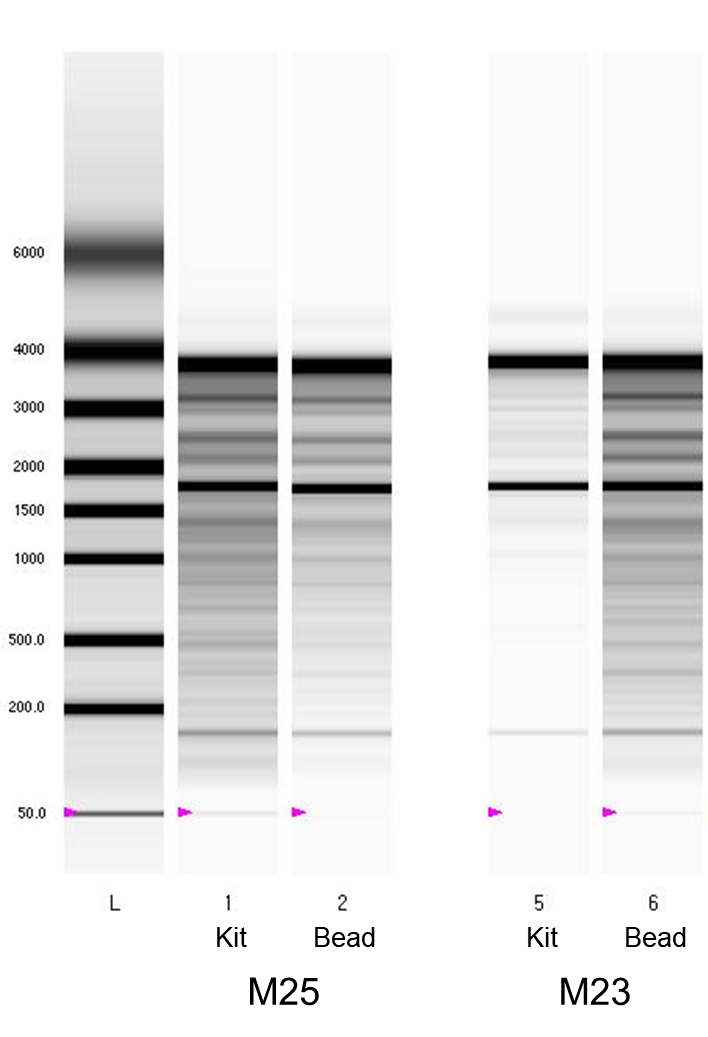
**

**Figure S3**. Representative gel-like images of exosome NA obtained from patients with malignant tumors. Exosomes were captured using aCD63-functionalized beads or ExoQuick kit. Note that aCD63-functionalized beads detected a 2.8-fold (p <0.001) more exosomes from cancer patients’ samples than non-cancer cohorts.


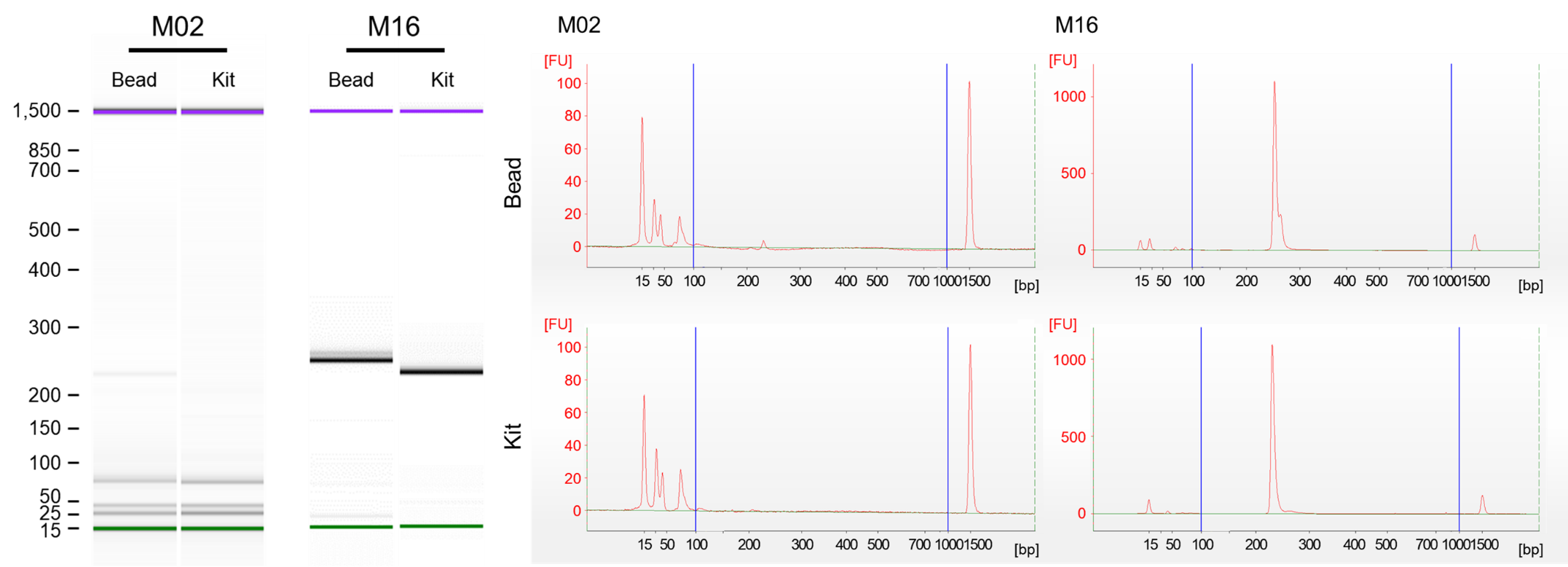


**Figure S4**. Representative electropherograms and gel-like images of cfDNA obtained from cancer. cfDNA was captured using PDA-SiO_2_-functionalized beads or QIAamp DNA mini-kit. Note that PDA-SiO_2_ beads detected a 12.2-fold (p <0.001) more exosomes from cancer patients’ samples than non-cancer cohorts.


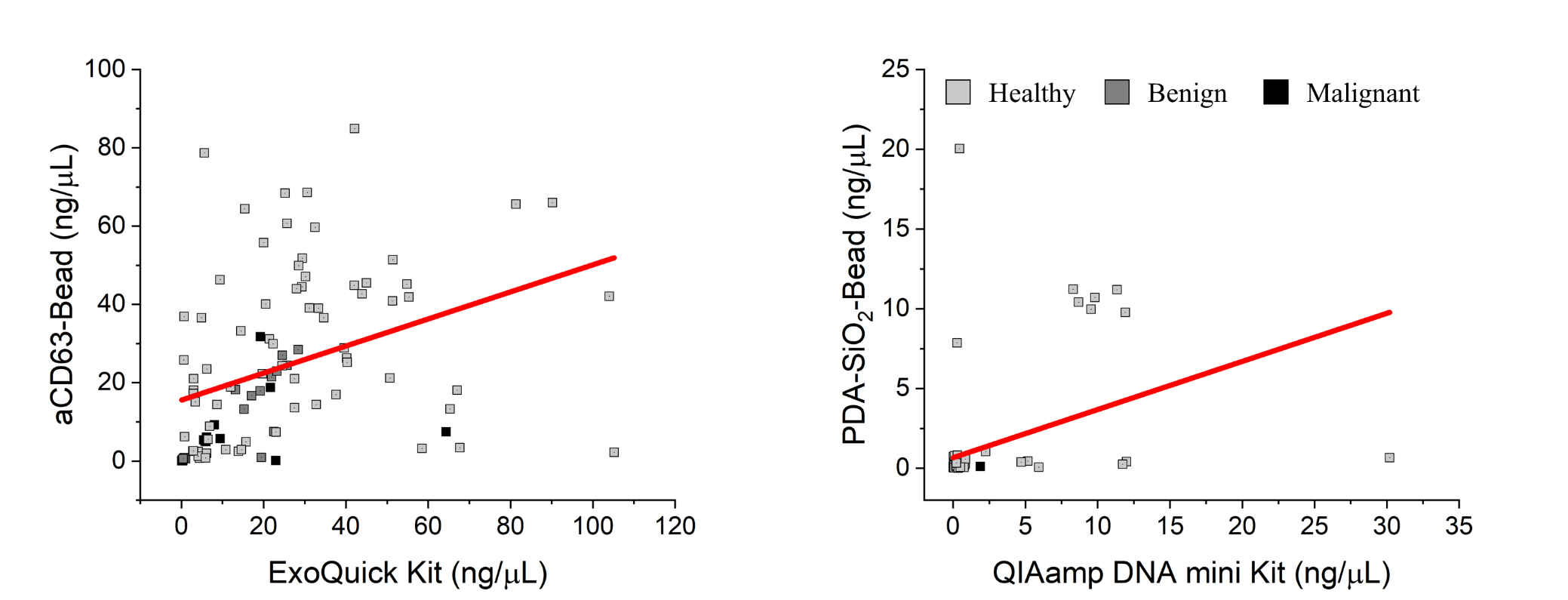


**Figure S5**. Pearson’s correlation analysis between the amounts of exosomes (left) and cfDNA (right) captured using the commercially available kits and the functionalized beads. The amount of exosome NA and cfDNA detected using the ExoQuick and Qiagen mini kit demonstrated a weak-to-moderate correlation with those detected using the bead-based systems, with Pearson’s coefficients (R) of 0.373 (exosomes; p < 0.001) and 0.385 (cfDNA; p< 0.001), respectively.

**
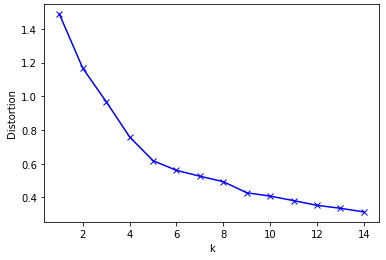
**

**Figure S6**. Elbow curve for the determination of optimal *k* in *k*-means clustering of the cohorts based on the expression levels of CTC, exosome, and cfDNA.


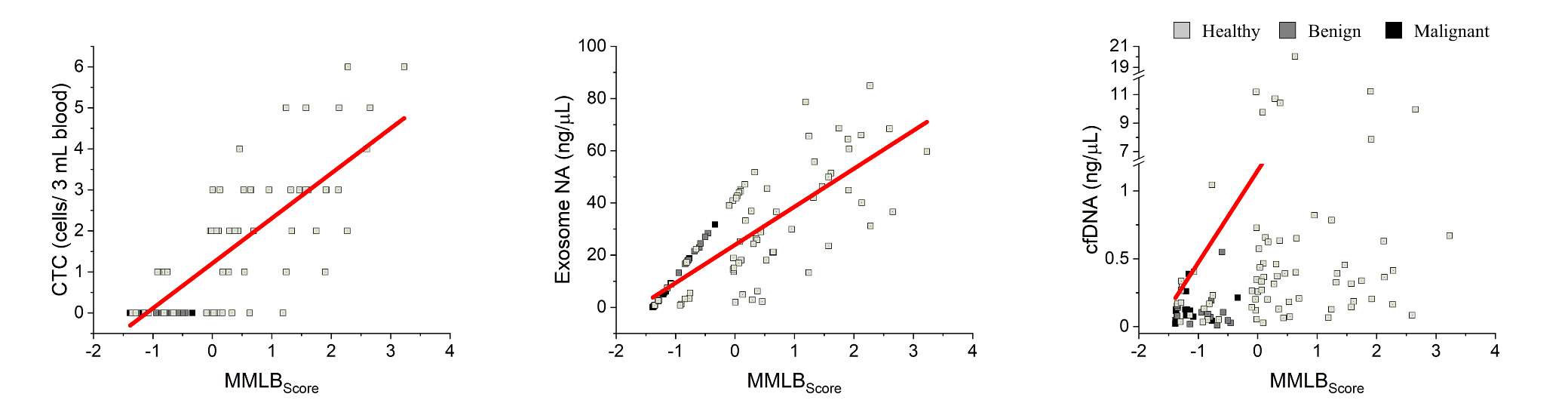


**Figure S7**. Pearson’s correlation analysis of MMLB_Score_ (PCA-X) with CTC (left), exosome (center), and cfDNA (right) for each patient.


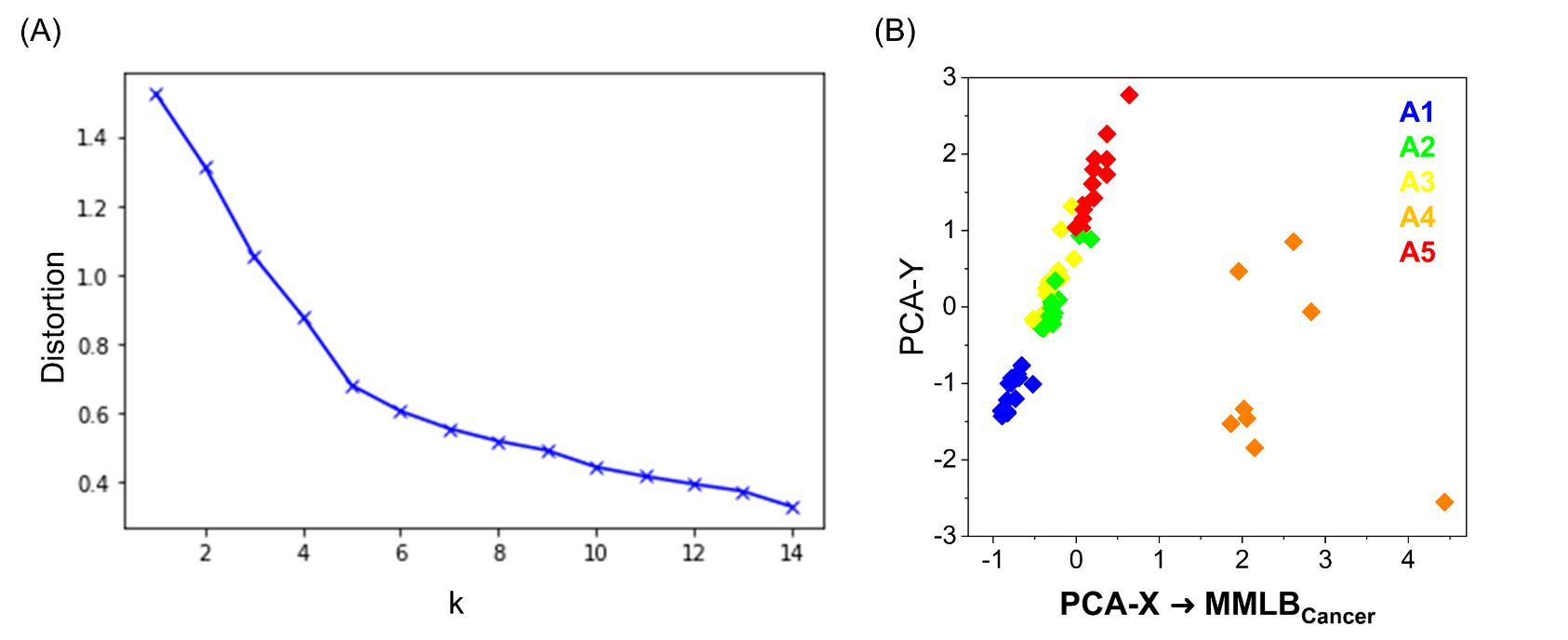


**Figure S8**. (A) Elbow curve for the determination of optimal *k* in *k*-means clustering of the cancer patients based on the expression levels of CTC, exosome, and cfDNA. (B) PCA applied to reduce the complexity of the 3D plot (the amount of CTC, exosome, and cfDNA) into an arbitrary 2D plot and provide the best linear approximation for stratifying the clusters for cancer patients. The elbow method and k-means clustering revealed that there were no differences in group assignments (A1-A5) for all 72 cancer patients compared to Figure 2B. However, PCA demonstrated the contribution of each biomarker to be different from the previous analysis, as the new MMLB_score_ (MMLB_Cancer_) exhibited a standardized coefficient of 0.188, 0.274, and 0.954 for CTC, exosomes, and cfDNA, respectively.


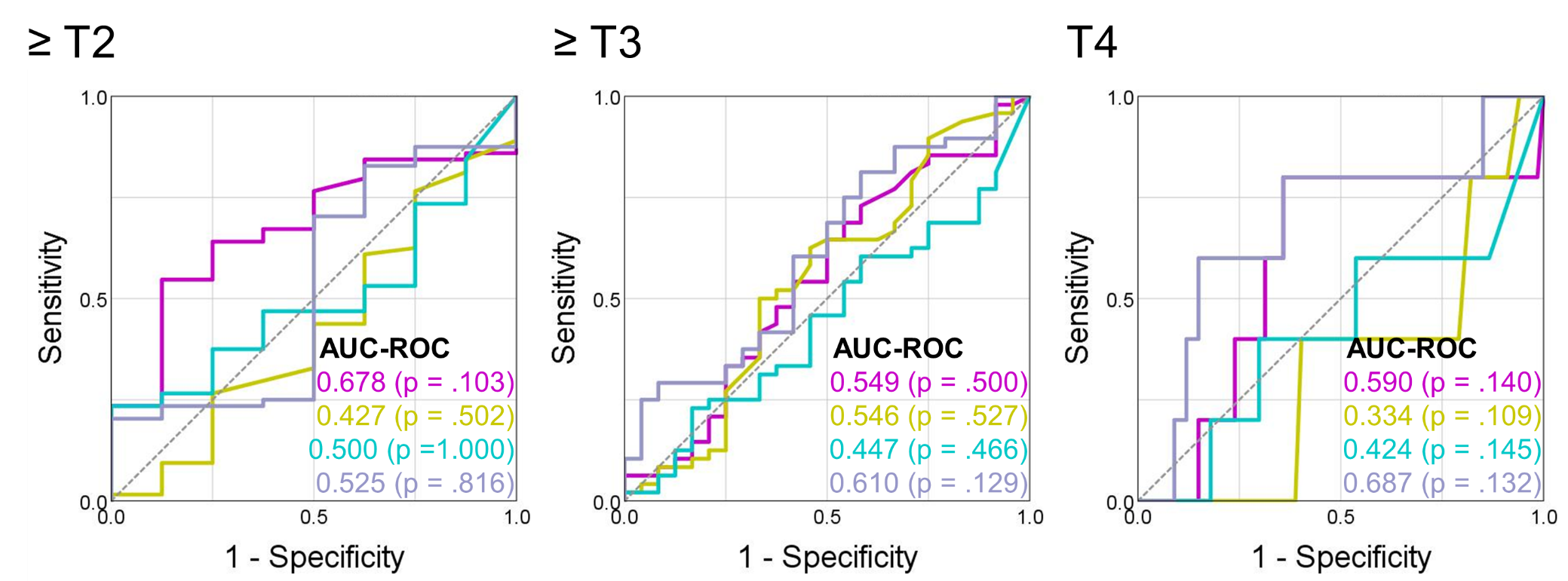


**Figure S9**. ROC curves demonstrating the diagnostic capability of serum antigens, including LDH (magenta), ADP (yellow), CA19-9 (cyan), and CEA (light blue), for differentiating advanced T stage patients.


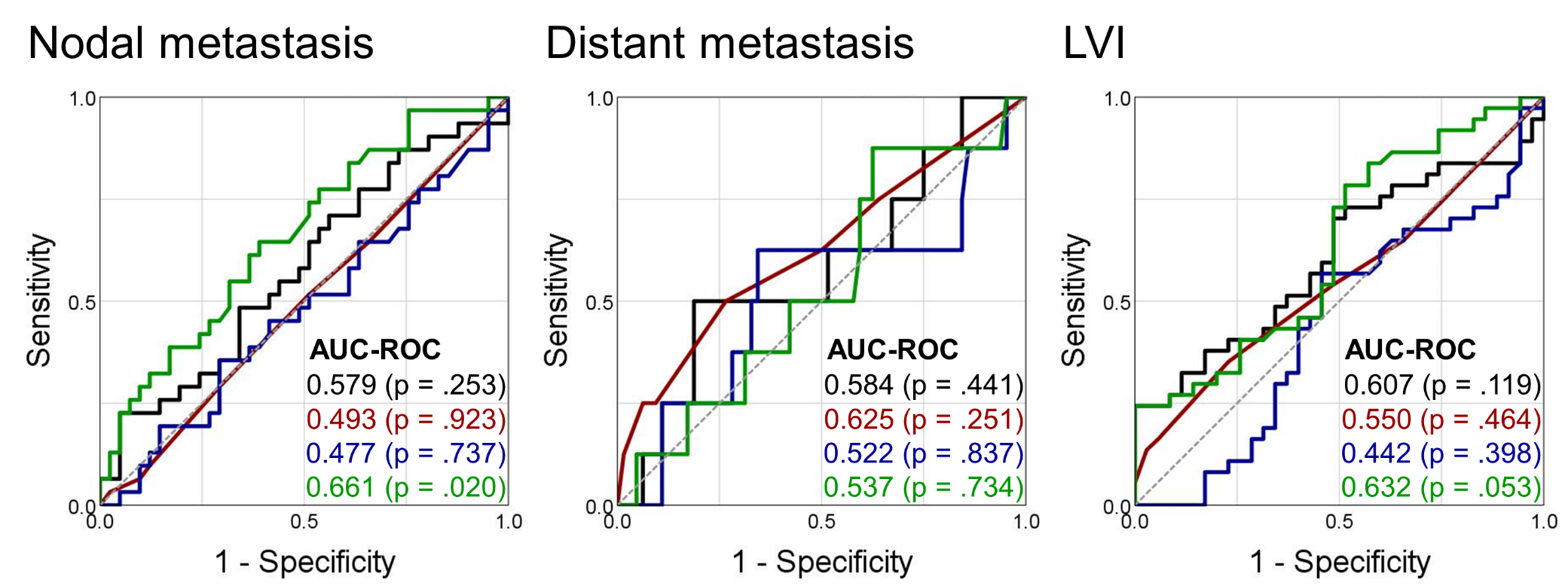


**Figure S10**. ROC curves demonstrating the diagnostic capability of MMLB_Cancer_ (black), CTCs (red), exosomes (blue), and cfDNA (green) for detecting LVI, nodal metastasis, and distant metastasis, compared to the single tumor biomarkers.


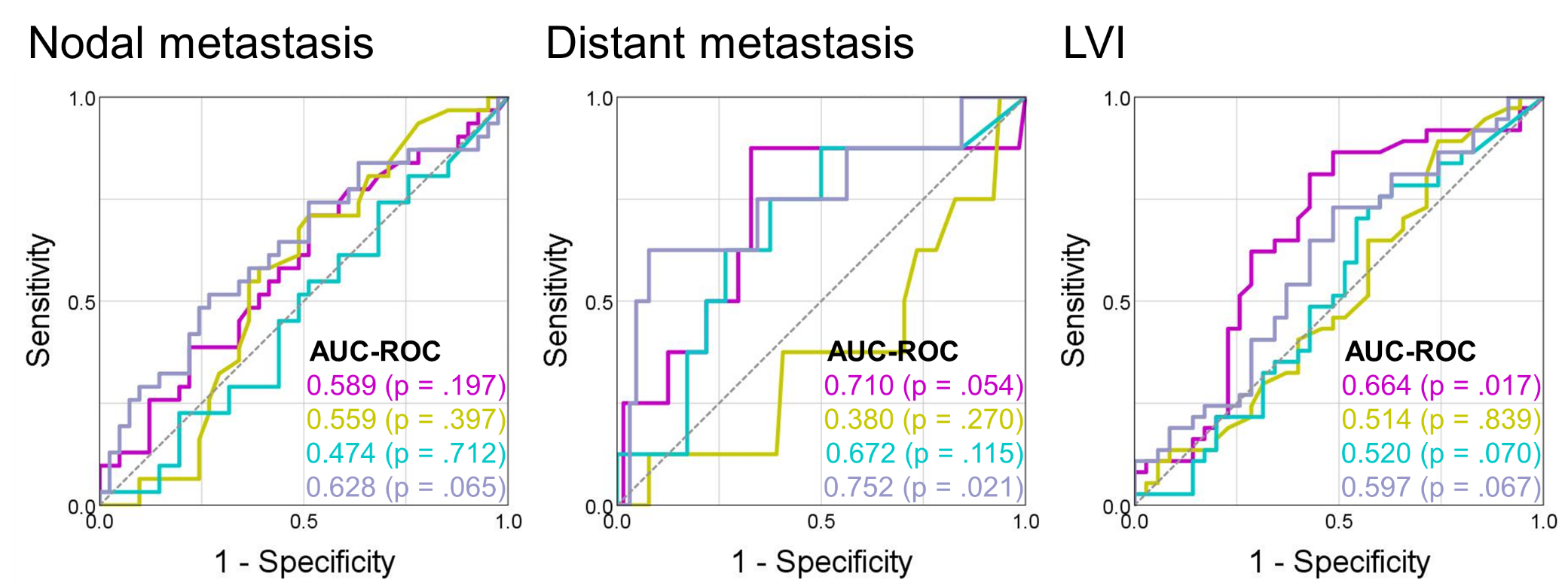


**Figure S11**. ROC curves demonstrating the diagnostic capability of serum antigens, including LDH (magenta), ADP (yellow), CA19-9 (cyan), and CEA (light blue), for detecting LVI, nodal metastasis, and distant metastasis, compared to the single tumor biomarkers.


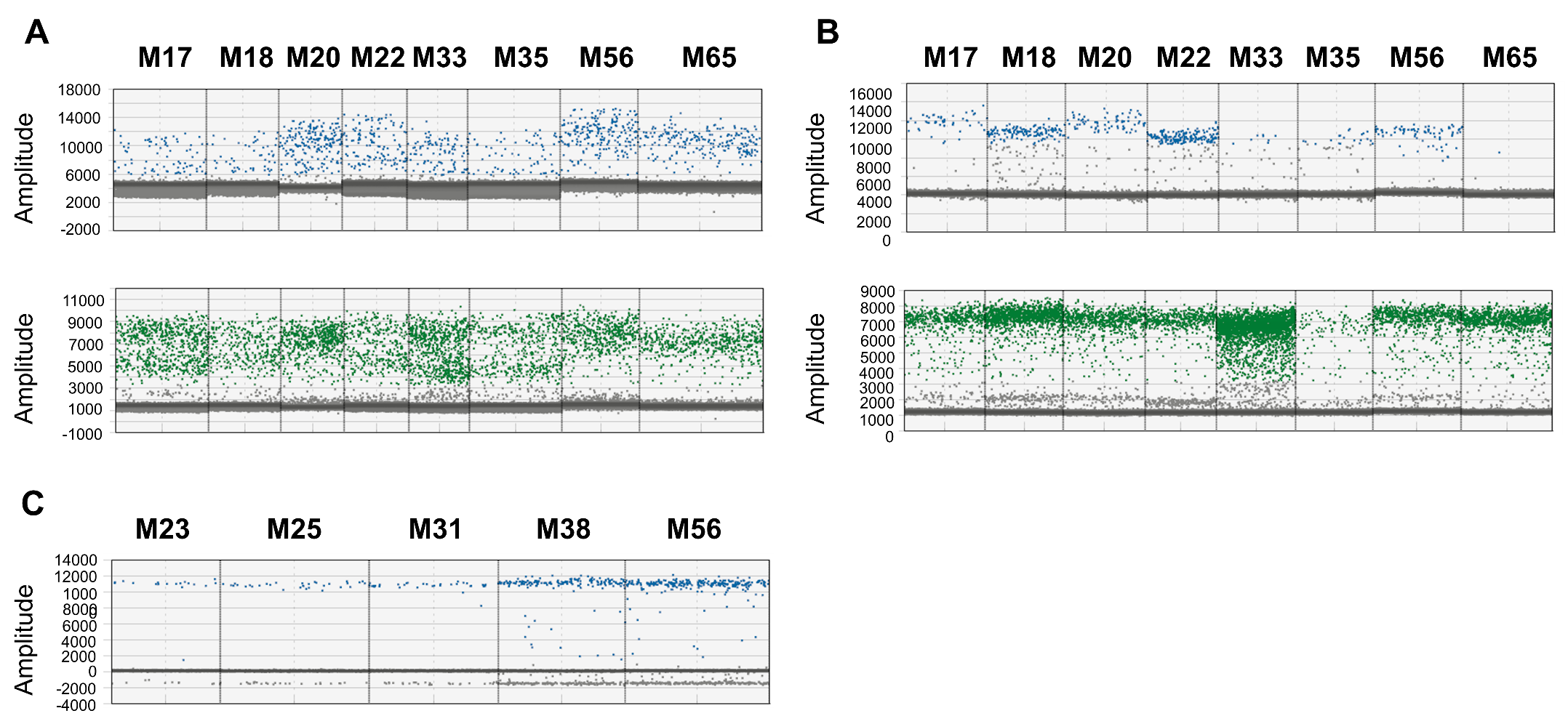


**Figure S12**. ddPCR analysis of (A) *KRAS* mutation in tissue (blue: mutant; green: wild type), (B) *KRAS* mutation in cfDNA (blue: mutant; green: wild type), and (C) miR-100 in exosome NA.

**
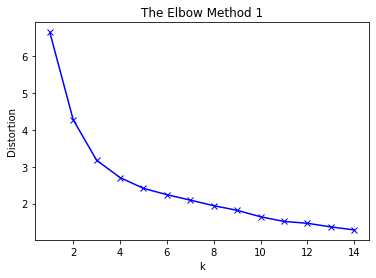
**

**Figure S13**. Elbow curve for the determination of optimal *k* in *k*-means clustering of the cohorts based on the expression levels of CTC, exosome miR-100, and *KRAS* MAF in cfDNA.


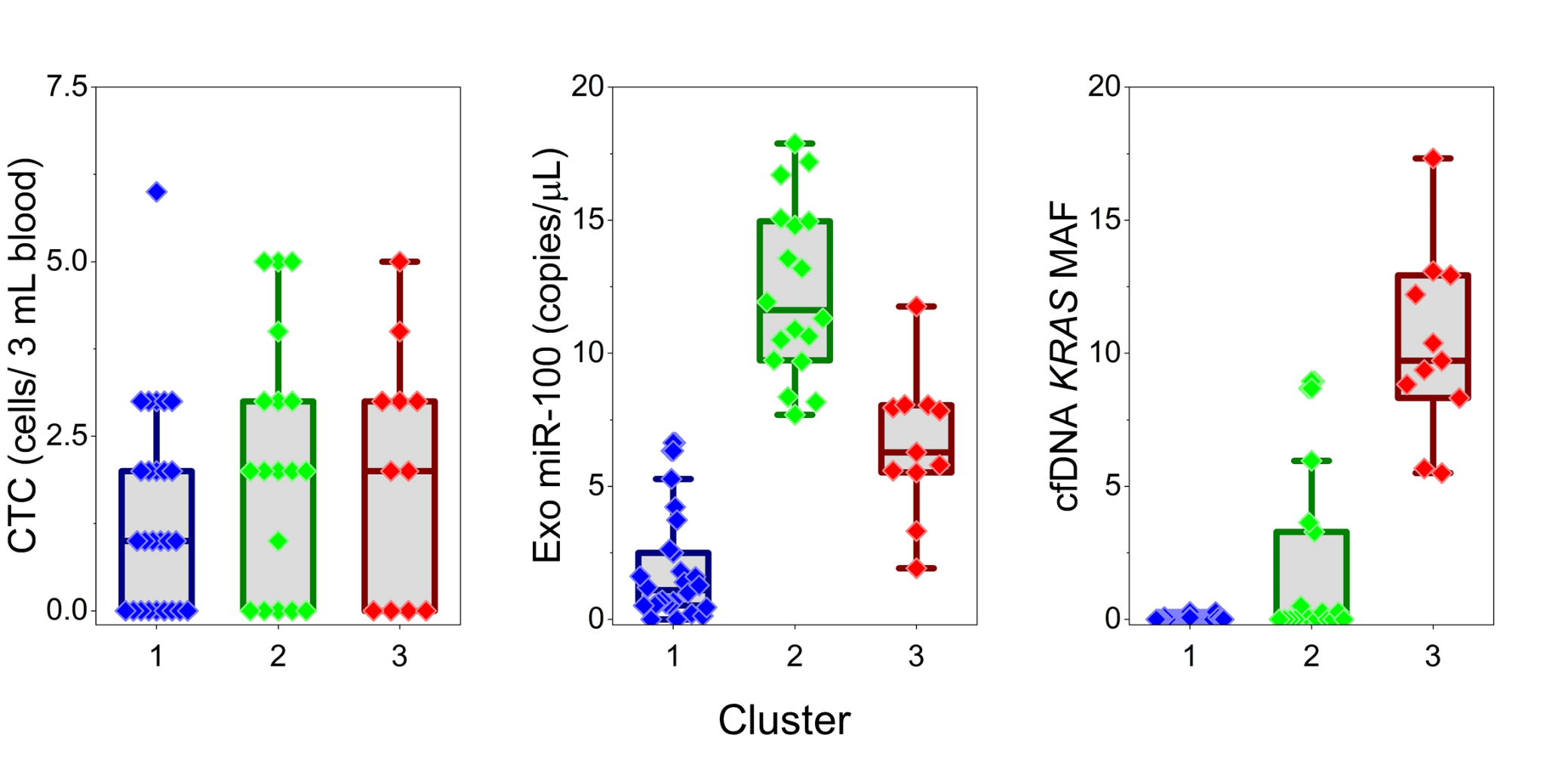


**Figure S14**. The difference in expression levels of CTCs (left), miR-100 (center), and *KRAS* MAF in cfDNA (right) between the clusters.


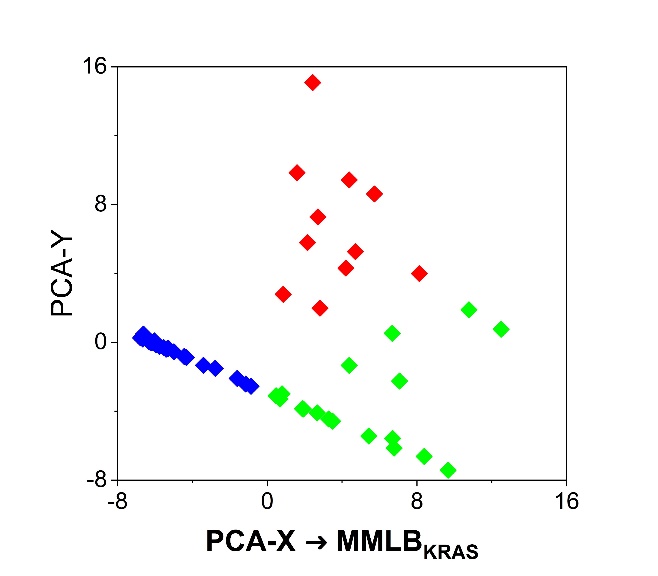


**Figure S15**. PCA applied to reduce the complexity of the 3D plot (CTC counts, exosome miR-100 expression, and *KRAS* MAF in cfDNA) into the arbitrary 2D plot which consisted of the two best linear approximations for stratifying the clusters.


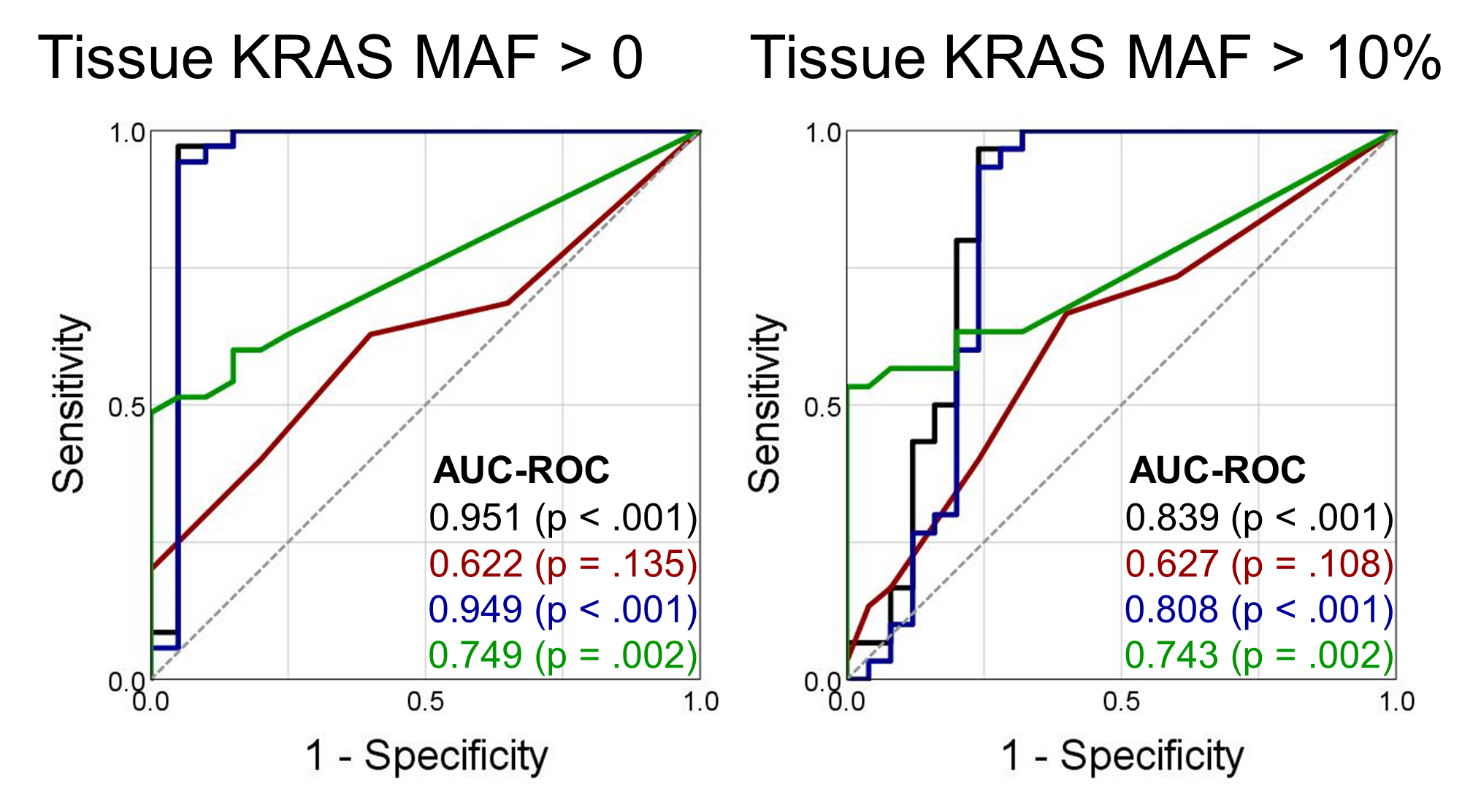


**Figure S16**. ROC curves demonstrating the capability of MMLB_KRAS_ for estimating KRAS mutation status in tumors compared to the single tumor biomarkers.
